# Global variation in the cost of a nutrient-adequate diet by population group: an observational study

**DOI:** 10.1016/S2542-5196(21)00285-0

**Published:** 2022-01-05

**Authors:** Yan Bai, Anna Herforth, William A Masters

**Affiliations:** a Friedman School of Nutrition Science and Policy, Tufts University, Boston, MA, USA; b Department of Economics, Tufts University, Boston, MA, USA; c Development Data Group, World Bank, Washington, DC, USA; d Food Prices for Nutrition Project, Boston, MA, USA

## Abstract

**Background:**

Nutrient deficiencies limit human development and could be caused by the high cost of locally available foods needed to meet nutrient requirements. We aimed to identify the populations whose nutrient needs are most difficult to meet with existing global food systems.

**Methods:**

In this observational study, we used the International Comparison Program 2017 collection of global food prices to measure cost per day and cost per calorie of meeting nutrient needs, based on least-cost diets within upper and lower bounds for energy and 20 nutrients for healthy populations across 20 demographic groups in 172 countries. We then analysed the composition of these least-cost diets by food groups to estimate how the affordability of foods for meeting nutrient needs varied by age, sex, and reproductive status.

**Findings:**

In 2017, the global median of diet costs per day was US$2·32 (IQR 1·95–2·76), with cost highest for adolescent boys aged 14–18 years at $2·72 (2·31–3·15). For females, median cost was highest for adolescents aged 14–18 years during pregnancy and lactation at $2·64 (2·29–3·15), exceeding the cost for adult men aged 19–30 years. The global median of diet cost per 1000 kcal was $0·94 (IQR 0·80–1·12), and was higher for females throughout the life course than for males, peaking for adolescent girls aged 9–13 years ($1·17 [95% CI 1·15–1·19]) and women older than 70 years ($1·18 [1·17–1·19]). Diet costs were most sensitive to requirements for calcium, iron, zinc, and vitamins C and E, as well as the upper bounds on carbohydrates and sodium. Total diet costs per day did not vary significantly with national income; however, in high-income countries, the composition of least-cost diets included more animal-source foods, whereas in low-income countries, diets with more pulses, nuts and seeds, and fruits and vegetables provided the most affordable way to meet nutrient requirements.

**Interpretation:**

Diets with adequate nutrients were unaffordable for many demographic groups, especially women and girls. These results could help to guide agriculture and food policy or transfer programmes to support populations at risk of inadequate intake.

**Funding:**

The Bill & Melinda Gates Foundation and UKAid.

## Introduction

Malnutrition is a multifaceted condition that limits human potential, and is often linked to insufficient or excessive intake of essential nutrients and other compounds. Malnutrition in low-income populations typically involves multiple deficiencies, often from diets high in starchy staples that do not have enough variety to ensure adequate intake of vitamins and minerals and balance across macronutrients.^1^, ^2^, ^3^, ^4^ Mortality attributed to micronutrient deficiency has decreased since 2007 but remains high,^5^ contributing to the development of diseases, deficits in human development, and losses in economic productivity,^6^ with great disparities across populations.^7^ High food prices can limit the ability of people to acquire diets with adequate nutrients,^8^, ^9^, ^10^, ^11^, ^12^ and many policies and programmes aim to improve diets by bringing nutritious foods within reach (ie, making them more affordable).^13^ Although it is well known that nutrient deficiencies are most prevalent among children, adolescents, and women who are pregnant or lactating, the cost and composition of complete diets that meet all nutrient requirements for each population has not yet been quantified globally.

Previous work on diet costs worldwide has focused on the needs of a single representative population group, such as adult women,^14^ or on the cost of adhering to a reference diet recommended for all people.^15^ To date, investigation of the variation in the cost of meeting requirements by age, sex, and reproductive status has been limited.^11^, ^12^ In this study, we aimed to identify how variation in nutrient needs affects diet costs globally, using the cost of nutrient adequacy (CoNA) based on the most affordable set of locally available foods that meet all requirements for essential nutrients in the target population. Our use of CoNA builds on a long tradition of least-cost diet calculations; in this case, to measure the ability of a food system to deliver nutrients in required amounts for each demographic group.^16^ Initial formulations of a least-cost diet used only lower bounds for a few nutrients, leading to unpalatable combinations of a few items;^17^ however, adding and updating nutrient requirements has allowed the method to be widely used to guide nutrition assistance and to improve poverty measurement.^18^, ^19^, ^20^

**Research in context**

**Evidence before this study**

Previous research on the most affordable foods to meet dietary needs found that costs exceeded available income for about 3 billion people (38% of the world population), with little systematic variation across countries but significant seasonal and spatial variation within Africa and south Asia. That research focused on dietary and nutrient requirements for a representative person, often an adult woman of reproductive age. Variation in the cost of meeting individuals’ needs by age, sex, and pregnancy or lactation was addressed only in studies at specific locations. The results and quality of previous research found that use of variants of the search term “diet cost nutrient adequacy” in PubMed, Europe PubMed Central, and Google Scholar depended on representativeness of the data for food prices, nutrient composition, and nutrient requirements. We found only one previous study that used nationally representative prices and all essential nutrients for the full range of demographic variation from early childhood to old age, including pregnancy and lactation, and it focused on a single country (Malawi).

**Added value of this study**

This observational study generalises previous findings to address demographic variation worldwide and quantifies global access to low-cost foods that can meet individual nutrient requirements for males and females at each stage of life, including pregnancy and lactation. The only age group we excluded was infancy (age <4 years), which is addressed in a companion paper through other methods. In this study, we focused on the least-cost way to meet requirements for a healthy person at the median of a global reference population in each demographic category, using food items for which availability and price had previously been reported by each country's national statistical organisation for other purposes. The least-cost diet approach isolates the effect of age, sex, and reproduction on access to nutrient adequacy, using widely available foods produced and delivered by national food systems. We found that affordability of a nutrient-adequate diet was influenced partly by the total amount of food needed in terms of dietary energy, which is highest for adolescent boys (aged 14–18 years) and women who are pregnant or lactating, but also by differences in nutrient requirements that lead to variation in costs per unit of dietary energy, which are highest for young adolescent girls (aged 9–13 years) and adult women older than 70 years.

**Implications of all the available evidence**

Global variation in access to low-cost diets that meet nutrient requirements by age, sex, and reproductive status points to opportunities for demographic targeting of food system interventions. Social protection and safety net programmes should be especially concerned about access to the more nutrient-dense items needed by adolescent girls, and the larger quantities of diverse foods needed during pregnancy and lactation. This evidence also shows which nutrients are least easily obtained through low-cost foods; for example, calcium for girls and women. Future work could build on these results to consider time constraints, cooking costs, and cultural or behavioural aspects of food choice, which impose additional barriers to the intake of nutrient-adequate diets.

## Methods

### Overview

In this observational study, we used global food price data for 2017 from the International Comparison Program (ICP) to measure the cost per day and cost per calorie of meeting nutrient needs in each country for healthy females from childhood and adolescence into adulthood, pregnancy and lactation, and old age, and for healthy males from childhood and adolescence into adulthood and old age. We analysed the composition of these least-cost diets by food groups to estimate how the affordability of foods for meeting nutrient needs varied among people by age, sex, and reproductive status. Our focus was limited to demographic variation in the least costly set of locally available foods that would achieve nutrient adequacy, recognising that any additional constraints, requirements, or recommendations would impose additional costs. Focusing on essential nutrients only allowed us to compute a lower bound on diet costs and to identify the influence of each requirement on the least-cost diet, indicating which nutrients were most expensive to obtain from foods commonly available in each country and the world as a whole.

### Food price and composition

Diet costs were based on national mean retail prices from 172 countries in 2017. The price for each food or beverage item in each country was released by the ICP in May, 2020, from data reported by national statistical agencies for standard items drawn from the ICP's global list of 193 foods and non-alcoholic beverages that could be sold worldwide, complemented by 523 additional items sold within specific geographical regions. From this list of 716 potentially available foods, each country reported a single, national mean price for the items that were actually available in 2017 at a nationally representative sample of diverse retail outlets in both rural and urban areas, typically visited every month or every 3 months throughout the year.^21^ The ICP reported prices for 175 countries and territories, but for this study we excluded three locations with missing income data (Anguilla, Bonaire, and Montserrat) that had a combined population of around 40 000 people in 2017. For reporting purposes, we grouped the remaining 172 countries by income level as classified by the World Bank into high income, upper middle income, lower middle income, and low income on the basis of their 2017 gross national income per capita.^22^ Price data provided by the ICP were in local currency units per unit of each food as purchased, which we converted into local currency units per kg. To find the edible portion and nutrient content of each item, we matched each product description to its corresponding entry in the US Department of Agriculture's National Nutrient Database for Standard Reference.^23^ A few items for which the edible portion and its nutrient contents were not listed in that food composition database—namely, some aquatic species and whole animals plus two plant foods (whole coconuts and cassava leaves)—were matched to regional and country food composition data.^24^, ^25^, ^26^ We excluded items that were non-caloric, as well as specialised infant foods, condiments consumed only in small quantities, and items with an unknown size or composition, resulting in a total of 545 items (appendix p 1). Each item's price was reported by a mean of 40 countries, and each country reported a mean of 125 prices, from a maximum of 261 items in Kazakhstan to a minimum of 37 items in Bermuda. We used these foods to identify the one least-cost diet for each demographic group in each country based only on nutrients and then, for reporting purposes, we classified items into six categories: starchy staples; pulses, nuts, and seeds; animal-source foods; fruits and vegetables; oils and fats; or sweets and beverages. All countries reported prices for at least one item in each of these categories, except for El Salvador, which did not report prices for the pulses, nuts, and seeds category (appendix p 4).

### CoNA

In this study, CoNA was defined as the least-cost diet meeting dietary reference intake (DRI) requirements for energy balance and 20 nutrients, including the upper and lower bounds for three macronutrients (ie, protein, lipids, and carbohydrates) and 17 micronutrients (ie, calcium, zinc, iron, magnesium, phosphorus, copper, selenium, sodium, vitamin C, thiamine, riboflavin, niacin, vitamin B6, folate, vitamin B12, vitamin A, and vitamin E; appendix p 9). The DRIs are from the US Institute of Medicine,^27^ and include upper and lower bounds from the acceptable macronutrient distribution range for total carbohydrates, protein, and fats; the upper level to avoid toxicity for 13 micronutrients; and an upper bound on sodium for chronic disease risk reduction (CDRR).^28^ For protein, carbohydrates, and all micronutrients, the lower bound in our main specification is the estimated average requirement (EAR) to meet the median level of need in each population. For iron and zinc, we applied higher harmonised average requirements^29^ to replace the EAR, reflecting the low bioavailability of these nutrients from least-cost diets, which are composed primarily of starchy staples, leguminous grains, and other vegetal foods. In a more restrictive scenario, we also applied the recommended dietary allowance (RDA) as the lower bound to meet or exceed the needs of 97·5% of each population.

Furthermore, for total estimated energy requirements, we used an active level of physical activity and the median weights and heights of WHO's reference population over 20 demographic groups, which included seven age categories (4–8 years, 9–13 years, 14–18 years, 19–30 years, 31–50 years, 51–70 years, and ≥71 years) for males and females, and requirements for pregnant or lactating women and girls in three age groups (14–18 years, 19–30 years, and 31–50 years).^30^

CoNA was defined as the solution to:

$$\min_{cg}({\text{CONA}}_{cg}=\sum_{i}P_{ic}\times q_{icg})$$

subject to:

$$\sum_{i}a_{ie}\times q_{icg}=EER_{g}$$

$$\sum_{i}a_{in}\times q_{icg}\geq {\text{LB}}_{ng}$$

$$\sum_{i}a_{in}\times q_{icg}\leq {\text{UB}}_{ng}$$

$$q_{icg}\geq 0$$

These equations allowed us to find the least-cost nutrient-adequate diet for each demographic group (g) in each country (c), using the least expensive set of food and beverage items (i) available on local markets at prevailing prices (p_ic_). The quantity of each item chosen for each group in each country (q_icg_) was just sufficient to meet their estimated energy requirements (EER_g_) and lower bounds (LB_ng_) or upper bounds (UB_ng_) for each nutrient (n), given each item's energy content (a_ie_) and nutrient composition (a_in_). For equation 3, there are lower bounds on the three macronutrients and 16 micronutrients—namely, calcium, copper, iron, magnesium, phosphorous, selenium, and zinc plus vitamins A, B6, B12, C, and E, as well as folate, niacin, riboflavin, and thiamine. For equation 4, there are upper bounds on all nutrients except for vitamin B12, riboflavin, and thiamine, and an upper bound on vitamin A in retinol form, and a CDRR upper bound for sodium. Because DRI requirements vary by demographic group, CoNA can be used to measure variation in the degree to which local food systems provide affordable diets, with all of the essential nutrients needed at every age and sex.

We calculated CoNA for each of the 20 demographic groups in all 172 countries, using linear programming to solve equations 1–4 for a total of 3440 different diets. Computations were first done in nominal local currency units and then converted into 2017 US$ by use of 2017 purchasing power parity conversion factors from the World Bank.^21^ We reported results as cost per day and, given that total quantity of food varied greatly by demographic group, we also reported cost per 1000 kcal to compare the cost of diet composition on an energy-adjusted basis. Our primary specification used EARs, reflecting the median level of requirement for a healthy population in each age-sex group. To address heterogeneity in requirements within each demographic group, we also reported results for all 3440 diets using RDAs, reflecting the 97·5th percentile to meet requirements for almost all healthy people in each group (appendix p 9). To test for significant differences among population groups, we regressed CoNA per day and CoNA per 1000 kcal on indicator variables for sex and age groups, and their interaction terms, adjusting for differences in national price levels using an indicator for each country.

### Sensitivity of diet costs to nutrient requirements

The definition of CoNA implies that, if each food had only one nutrient, every nutrient listed in equations 2 and 3 would have its own least-cost source of that nutrient, and equation 1 would simply add up the total costs of those foods. In practice, each food provides multiple nutrients, and mathematical programming was needed to compute the most cost-effective combination of available food items to meet all nutrient requirements. At the least-cost solution, the number of foods equals the number of constraints that are binding. The incremental difference in CoNA caused by small changes in each nutrient requirement shows the degree to which locally available foods complement each other to meet human needs at low cost. The level of incremental cost associated with each nutrient is known as the shadow price of each constraint, measured in terms of the currency units of equation 1 per unit of each nutrient in the relevant constraint from equations 2, 3, or 4. Non-binding constraints have a shadow price of US$0. To show variation in the sensitivity of diet costs to each nutrient requirement across demographic groups, we focus on percentage changes and report each constraint's shadow price elasticity, as defined by:

$$e_{sp}^{j}=\frac{\%\Delta \text{CoNA}*}{\%\Delta {\text{DRI}}_{j}}$$

where the elasticity (e^j^_sp_) of the shadow price (sp) for each constraint (j) is the percentage change in the least-cost solution (%ΔCoNA*) for a 1% change in that requirement. A high energy requirement specified in equation 2 not only calls for an increased quantity of food in calorie terms that might increase costs (indicated as a positive *e*_sp_), but also allows for use of foods with low nutrient density per calorie, which might have low prices that could reduce diet costs and lead to a negative *e*_sp_. For each individual nutrient, costs would rise with higher levels of lower-bound requirements shown in equation 3, and costs would decrease with higher levels of upper-bound limits in equation 4. To compare the importance of each constraint, we showed the magnitude of shadow price elasticities in terms of absolute value.

### Variation in cost by demographic group within countries

To quantify differences in diet costs across demographic groups, we showed the full distribution of diet costs for each group using box plots. We then computed the difference in least-cost diets caused by variation in requirements across groups using general linear models. The regressions focused on the marginal effect of mean differences in age, sex, pregnancy, and lactation, across all countries, adjusted for each country's mean diet costs. The inclusion of an indicator variable for each country's fixed effects assimilated all cross-country differences associated with the national food systems described in other studies.^14^ This approach also accounted for differences in price reporting, such as the artifactually high level of CoNA in countries that use short food lists, omitting items that would otherwise be included in least-cost diets (appendix p 2).

### Computational and statistical methods

We conducted linear programming using the lpSolve package in R,^31^ and presented results using box and whisker plots presenting the median, 25% quantile, and 75% quantile for each variable, as well as the range of median (1·58 × IQR/N^1/2^).^32^ We estimated variation in CoNA per day and CoNA per 1000 kcal associated with each demographic characteristic using general linear models for sex, age groups, and their interaction terms, controlling for countries’ fixed effects to account for any geographical variation. We also compared food quantities in the least-cost diets across the six major food categories, and computed shadow price elasticities for the nutrients that were most often binding across all demographic groups, as well as the differences in CoNA associated with a country's level of national income. We used Stata (version 15.1) and R (version 4.1.0) for computation, data processing, statistical analyses, and visualisations.

### Role of the funding source

The funders of the study had no role in study design, data collection, data analysis, data interpretation, or writing of the report.

## Results

CoNA per day for all demographic groups across 172 countries in 2017 had a global median of US$2·32 (IQR 1·95–2·76). Adolescent boys aged 14–18 years had the highest cost, with a median of $2·72 (2·31–3·15), followed by lactating adolescent girls of the same age group, with a median of $2·64 (2·29–3·15). Across countries of different income levels, middle-income countries faced the highest CoNA, with a median of $2·56 (2·18–2·99) for upper-middle-income countries and of $2·39 (2·03–2·80) for lower-middle-income countries (appendix p 10). When the level of nutrient requirements was changed from EARs to RDAs (ie, from meeting the needs of 50·0% to 97·5% of a healthy population), the median CoNA increased by 17·2% to $2·72 (2·31–3·24) with little change in the patterns across demographic groups and country income levels (appendix p 11).

**Figure 1:**
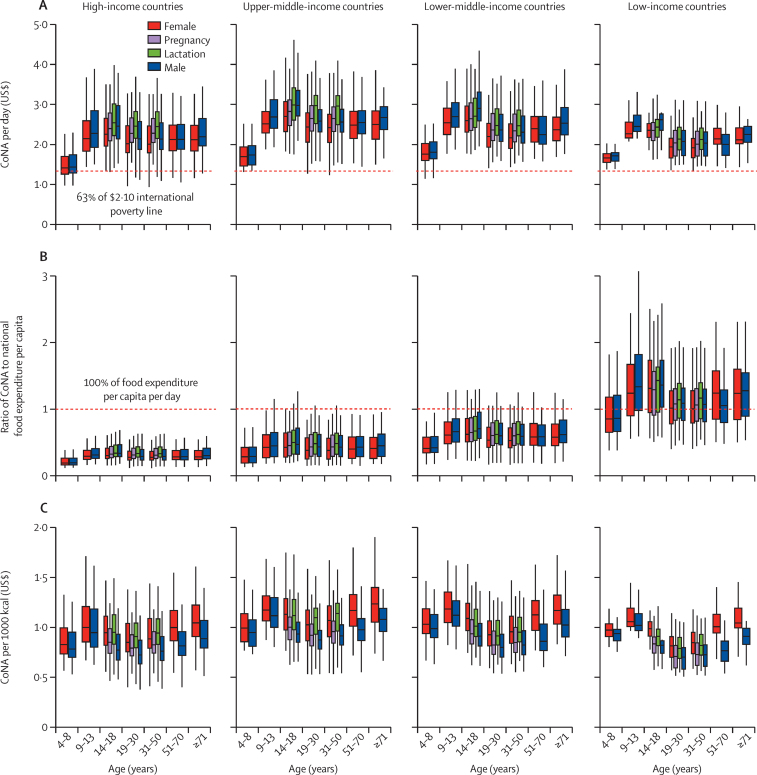
CoNA for 20 demographic groups across 172 countries in 2017 by national income category (A) The CoNA in US$ per day reflects the range of cost per day in 2017 for sufficient quantities of the most affordable foods available in each country to meet daily nutrient requirements for a healthy population in each demographic group. The horizontal dashed line facilitates comparison to a global poverty line with food expenditure levels of $1·32 per day, corresponding to 63% of $2·10 per day in expenditure on all goods and services in 2017. (B) The ratio of CoNA to national food expenditure per capita reflects the range of ratios between the least-cost diet and the observed quantity of food expenditure per capita per day in 2017, using the total annual food expenditure in the International Comparison Program 2017 divided by the 2017 population and 365 days. The horizontal line at 1 refers to the country's actual mean food expenditure per capita. (C) The CoNA per 1000 kcal reflects the range of diet costs per 1000 kcal for sufficient quantities of the most affordable foods available in each country to meet median daily nutrient requirements for a healthy population in each demographic group. Vertical lines from the box plots show the range of median (1·58 × IQR/N^1/2^). CoNA=cost of nutrient adequacy.

In 2017, median CoNA per day was typically above the international poverty line of US$2·10 (figure 1A) and above actual food expenditures in low-income countries (figure 1B).^32^, ^33^ In all cases, median CoNA was above $1·32 per day, which is 63% of the poverty line income that can be credibly reserved for food expenditures.^11^ The pattern of cost by demographic group was similar at each level of national income. Adjusting for differences in energy consumed by people in each demographic group, median CoNA per 1000 kcal was at least $0·94 (0·80–1·12) when using EARs and at least $1·10 (0·94–1·31) when using RDAs (appendix pp 12–13). Variation in the cost of required nutrients per 1000 kcal by age over the life course is N shaped, with the highest costs for adolescents aged 9–13 years and adults aged 51 years or older who require more nutrient-dense diets (figure 1C). Females of all ages faced higher median costs per 1000 kcal than did males (figure 1C).

**Figure 2:**
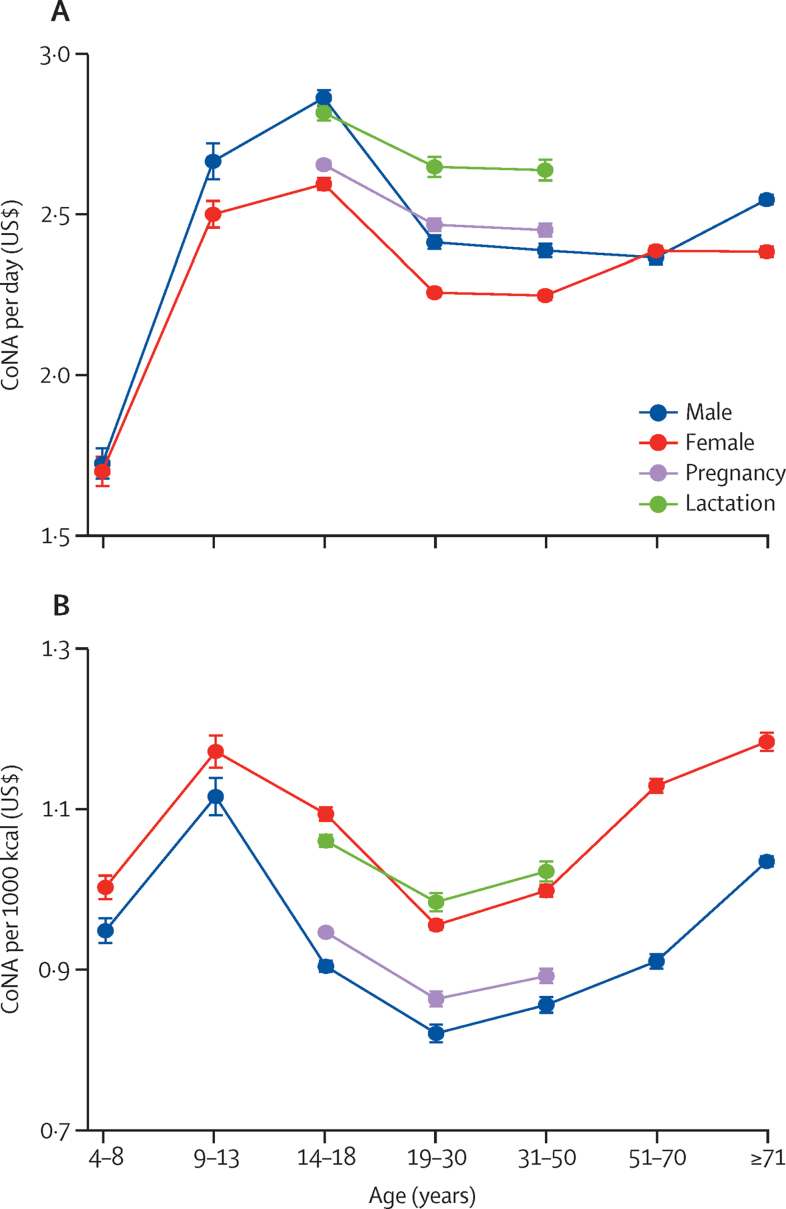
Variation in mean diet cost among demographic groups CoNA per day (A) and per 1000 kcal (B). All data shown refer to the least-cost diet meeting all nutrient requirements for the median person in a healthy population from each group. Error bars show 95% CI around the mean for each group, from a regression with the fixed effects of 172 countries that controlled for each country's differences in mean costs for all demographic groups. CoNA=cost of nutrient adequacy.

Regression results showed variation in CoNA caused by differences in requirements between each demographic group, controlling for mean diet costs in each country (figure 2). Among all groups, mean diet cost per day peaked at $2·87 (95% CI 2·84–2·89) in adolescent boys aged 14–18 years. Lactating adolescent girls aged 14–18 years faced a high mean CoNA per day of $2·82 (2·80–2·85). For males and females, the mean CoNA per day of adolescents aged 14–18 years and adults older than 70 years were also significantly higher than that of adults aged 19–30 years.

Younger children (aged 4–8 years) had a significantly lower mean CoNA than did other age groups due to lower energy needs (appendix p 14). Regarding the energy-adjusted costs per 1000 kcal, the highest estimates were found for young adolescent girls (aged 9–13 years; $1·17 [1·15–1·19]) and adult women older than 70 years ($1·18 [1·17–1·19]; figure 2B). Females in all age groups had significantly higher energy-adjusted costs than did age-matched males, and younger people (aged <18 years) and adults older than 50 years had a significantly higher CoNA per 1000 kcal than did adults aged 19–30 years (appendix p 14).

**Figure 3:**
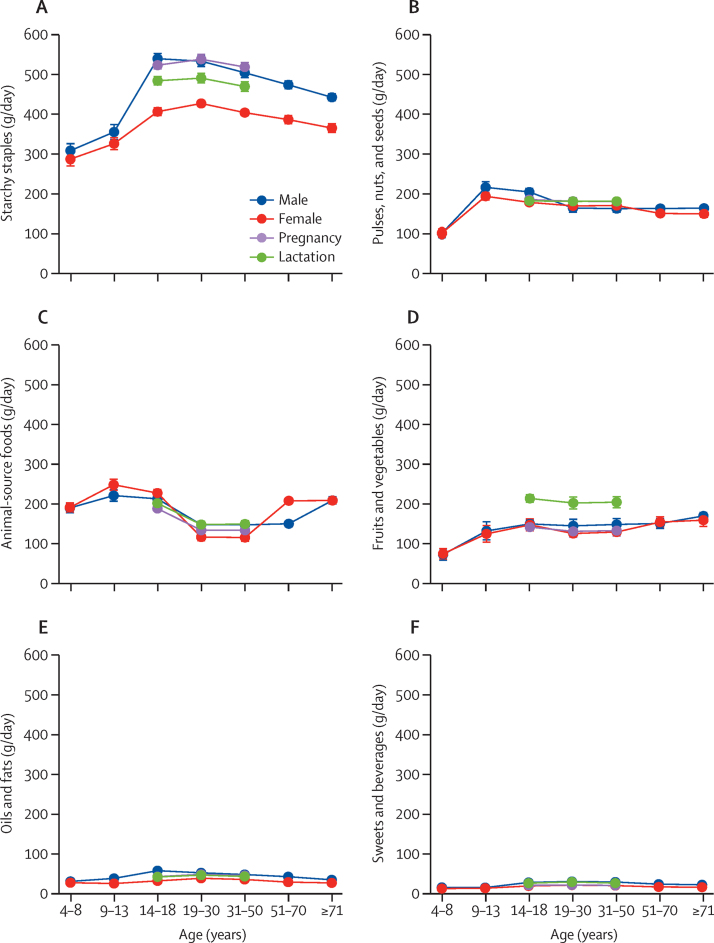
Composition of least-cost diets by food category and demographic group Quantities per day of starchy staples (A); pulses, nuts, and seeds (B); animal-source foods (C); fruits and vegetables (D); oils and fats (E); and sweets and beverages (F) that meet all nutrient requirements in a least-cost diet for the median person in a healthy population from each group. Error bars show 95% CI around the mean for each group, from a regression with the fixed effects of 172 countries that controlled for each country's differences in mean costs for all demographic groups.

The most affordable diets to meet all nutrient requirements used large quantities of starchy staples, complemented by a mixture of nutrient-dense foods. Over all demographic groups, least-cost diets included an average of 438 g of the most affordable local starchy staples, providing 1281 kcal per day or about 44% of total weight and 52% of total dietary energy (appendix pp 15–16). Foods selected for the least-cost diets depended on availability and price in each country, but included a mixture of all major food groups as shown for ten example countries in the appendix (p 3). With respect to variation in diet composition by demographic group, least-cost diets for people aged 14–50 years included a much larger volume of starchy staples than did those for individuals younger than 14 years or older than 50 years (figure 3A). Most of the few animal-source foods included were dairy products, accounting for 85% of the weight and 79% of the energy of animal-sourced foods that appeared in least-cost diets, primarily for young adolescents (aged 9–13 years) and adults older than 50 years. By contrast, significantly more fruits and vegetables were selected to meet the nutrient needs of lactating women of all age groups than for all other population groups. The least-cost diets in low-income countries included 138 g/day less animal-source foods than in high-income countries, and 146 g/day more pulses, nuts, and seeds, as well as 81 g/day more fruits and vegetables, to meet the same set of nutrient requirements on average, after adjusting for differences by demographic group (appendix p 17).

**Figure 4:**
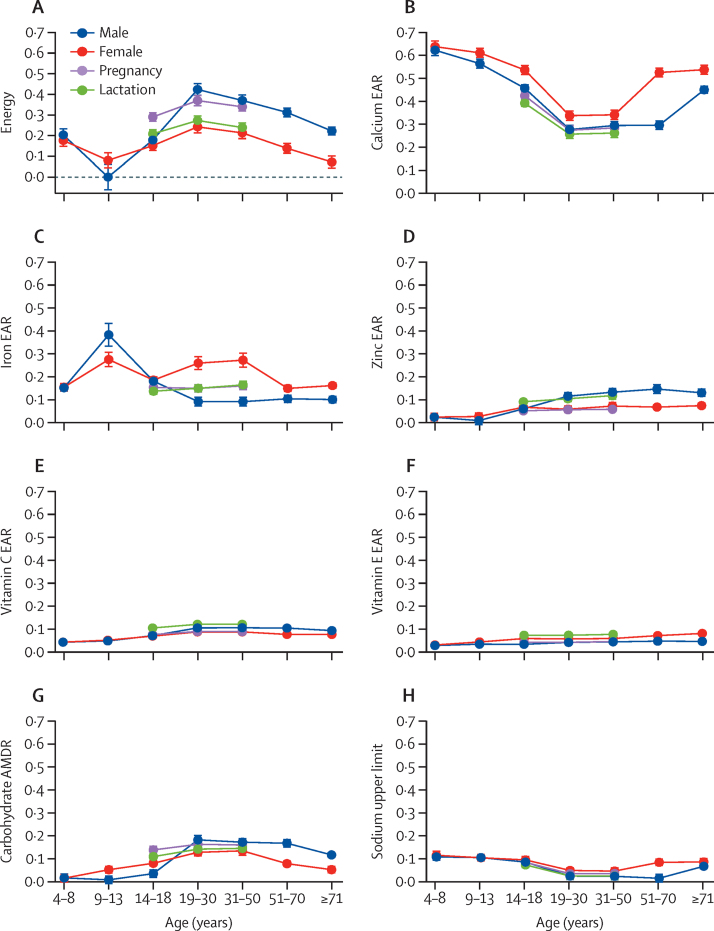
Sensitivity of diet costs to selected nutrient requirements by demographic group Shadow price elasticities, defined as the percentage change in diet cost for each 1% change in the nutrient requirement shown, for the eight most important constraints: energy (A); the EARs for calcium (B), iron (C), zinc (D), vitamin C (E), and vitamin E (F); the upper bound limit (ADMR) of carbohydrates (G); and the upper bound limit of sodium for chronic disease risk reduction (H). For comparability purposes, we used the magnitude (absolute value) of shadow price elasticities for the upper bound limits of carbohydrate and sodium. Error bars show 95% CI around the mean for each group, from a regression with the fixed effects of 172 countries that controlled for each country's differences in mean costs for all demographic groups. EAR=estimated average requirement. AMDR=acceptable macronutrient distribution range.

The nutrient requirements for which diet costs were the most sensitive were total energy; lower bound requirements for calcium, iron, and other micronutrients; and upper bound limits on carbohydrates for macronutrient balance (appendix p 18). Across demographic groups, meeting calcium requirements was particularly expensive for children, adolescents, and adults older than 50 years, and meeting iron requirements was particularly expensive for young adolescents, especially boys aged 9–13 years and for non-pregnant and non-lactating women aged 19–50 years (figure 4). For adults, especially men older than 18 years, diet costs were more sensitive to total energy requirements and to the upper bound on carbohydrates. Least-cost diets were slightly more sensitive to zinc requirements among adult men older than 50 years than among age-matched women, and more sensitive to vitamin C and E requirements among lactating females of all age groups than among all other groups. Vitamin E had a somewhat larger role in requirements for adult women older than 50 years, and diet costs for children and adolescents were somewhat more sensitive to the sodium CDRR. Diet costs were the most sensitive to calcium requirements in low-income countries, perhaps due to the high cost of dairy products. Other differences in the cost and composition of available foods increased the sensitivity of diet costs to the sodium CDRR in low-income and middle-income countries, and of the acceptable macronutrient distribution range for carbohydrate in middle-income countries (appendix p 19).

## Discussion

To our knowledge, this study is the first to quantify variation in the cost of meeting nutrient requirements by demographic group in the global population for males and females from childhood to old age, including periods of pregnancy and lactation. Previous work on demographic variation in the cost of meeting a complete set of nutrient requirements focused on a single country, Malawi, where a limited range of foods were available.^12^ In this study, we compared diet costs for each demographic group using food items available in each of 172 countries, representing almost all of the world's national food systems, and found three main results.

First, we extend previous observations that meeting nutrient needs is often unaffordable.^11^, ^14^ We found that least-cost diets for nutrient adequacy, even without considering the additional cost of meeting other constraints related to diet quality, meal preparation, and food preferences, would exceed income per capita of anyone below global poverty lines in every one of our demographic groups and in all regions of the world. The median cost per day across countries was highest for adolescents aged 14–18 years at US$2·72 for boys and $2·44 for girls, plus an additional $0·05 during pregnancy and $0·20 per day during lactation. Diet costs are lowest for children aged 4–8 years, at $1·67 for boys and $1·64 for girls. These costs exceed what households at the international poverty line can spend on food, an average of 63% of all expenditure (or $1·32 per day),^11^, ^14^ thereby confirming that increasing income, reducing prices, or offering some form of external assistance would be needed for these individuals to obtain adequate nutrients at any stage of life. Affordability is a necessary but not sufficient condition for people to choose nutrient-adequate diets. Computing least-cost diets shows that, in low-income settings, nutrient-adequate diets are often unaffordable. Using least-cost diets as a benchmark allows us to distinguish between unaffordability and other barriers to consumption.

Second, we found significant variation in least-cost diets across demographic groups. Focusing on differences in nutrient density, costs per 1000 kcal were higher for females than for males at any age, and were highest for adolescent girls (aged 9–13 years) at $1·17 and for adult women older than 70 years at $1·18. These findings are well above the global median diet cost of $0·94 per 1000 kcal due to the need for a high density of the nutrients that are most expensive to obtain from the foods available in each country. The nutrient requirements to which diet costs were most sensitive were calcium and iron, followed by zinc and vitamins C and E. Many micronutrient supplementation programmes include iron and zinc, and sometimes calcium. Our results add evidence to verify that these nutrients are particularly out of reach for many people, such as iron for women of reproductive age. Diets that meet other nutrient requirements are generally adequate in vitamin A at little or no additional cost (appendix p 18), even in low-income countries, suggesting that having a nutritious diet overall could also prevent vitamin A deficiencies. Furthermore, we found that keeping diets below the upper bound on sodium is an important driver of total cost, particularly for younger and older groups and for populations in low-income countries. This finding suggests that otherwise balanced diets can easily have excess sodium, given the nutrient composition of food items available in national markets.

Third, we found that meeting nutrient needs at the lowest cost required a different mixture of foods for each demographic group, and that this pattern varied across countries. Least-cost diets for adolescents, lactating women, and older people included more nutrient-dense foods (eg, pulses, nuts, and seeds; animal-source foods; and fruits and vegetables) and fewer starchy staples than would be required to meet nutrient requirements for adult men (aged 19–70 years). Lowering the cost of these foods through innovations in agricultural production, trade, and distribution that reduce market prices could help to reduce inequities in diet costs across sex-age groups. Another kind of inequity exists between countries, whereby the high prices for dairy and eggs in low-income countries imply that the most affordable mixture of foods to meet nutrient requirements includes less animal-source foods and correspondingly more pulses, nuts, and seeds, as well as fruits and vegetables, than do the most affordable diets in high-income countries. This substitution between food groups to meet DRI requirements raises concerns about how other aspects of healthy diets, as specified in dietary guidelines, would be affected by differences in cost among food groups.^11^, ^15^ The observation that some animal-source foods were less costly in high-income countries than in low-income countries might have a role in driving food choices away from plant-source foods that are recommended in dietary guidelines and for global sustainability goals.

One limitation of this study is having only one national mean annual price for each food in each country. More detailed data from within countries would be needed to study temporal or spatial variation. Additionally, the list of foods for which market prices were collected does not represent all of the items and ways to obtain food that could potentially be used in low-cost diets. If local food environments actually offered low-cost ways to obtain required nutrients, this dataset would have overstated the degree to which it was unaffordable for people to obtain nutrient-adequate diets. The ICP provides the most comprehensive collection of internationally standardised data on food prices; however, without prices for every food in every location, it remains possible that low-income consumers could obtain required nutrients at a lower cost than our results suggest. Reduced cost options might include home production, gifts, or purchase of less attractive versions of the items included in this study, or entirely different items, such as bushmeat or wild fruits at particular places and times. Other studies that addressed variation within countries, including the cost of resources used for home production, found cost per day estimates of similar magnitude to our results, but with substantial spatial, seasonal, and other variation over time, which is subject to further research.^34^ A further limitation concerns use of a single estimate of nutrient composition for each food item, primarily from the US Department of Agriculture's National Nutrient Database for Standard Reference data, whereas future work should address variation and uncertainty in how much of each nutrient is in each food at each location, and the degree to which those nutrients are absorbed due to interaction among foods and other determinants of bioavailability for each nutrient. In this observational study, we conducted the analysis using higher nutrient recommendations (harmonised average requirements) that assumed lower bioavailability of zinc and iron; however, additional data on food composition and absorption of nutrients, as well as improvements in estimated nutrient requirements, would be needed for greater accuracy. Additionally, by design, the purpose of CoNA is to quantify the cost at market prices of acquiring sufficient quantities of locally available food items to meet a population's nutrient requirements. CoNA provides a lower bound on total costs, as a first step towards quantifying affordability. When accounting for the fact that people do not generally know the nutrient contents of each food or their dietary requirements, a more behaviourally feasible set of criteria towards meeting nutrient needs is the cost of meeting food-based dietary guidelines, in the Cost of a Healthy Diet metric.^11^ Additional costs of meal preparation to be addressed in future studies would include time use, cooking fuel, water, and sanitation for food safety, among many other factors.^35^ Furthermore, our analysis concerns only the access dimension of food production and distribution, using the affordability of least-cost diets to measure whether food systems bring nutritious items within reach for every demographic group. Where and when healthy diets are affordable, and the healthiness of the products that people actually consume depends on many other factors, including food preferences and food allocation within households.

Our study shows that the worldwide cost of nutrient-adequate diets varies by demographic group, but exceeds available income for all people living in extreme poverty. The cost per day of reaching nutrient adequacy would be highest for adolescents and for women who are lactating, and diet composition in terms of cost per calorie would be the most expensive for females of all ages, particularly over age 50 years. We found that one of the most expensive food groups that make diets unaffordable are nutrient-dense plant foods (eg, fruits and vegetables or pulses, nuts, and seeds), leading to high costs of obtaining sufficient amounts of calcium, iron, and other nutrients. Targeted intervention could reduce the cost of these limiting factors, thereby helping to ensure that nutrient-adequate diets are affordable to meet the needs of all demographic groups worldwide.

## Data sharing

ICP-2017 global price data are available on request under confidentiality agreements with the ICP team of the World Bank. Price index data and the model code will be posted at the project website.

## Declaration of interests

We declare no competing interests.
