# Supplementary material for: Global variation in the cost of a nutrient-adequate diet by population group: an observational study
Source: Lancet Planet Health. 2022 Jan 5;6(1):e19–28. doi: 10.1016/S2542-5196(21)00285-0 (PMC8753783; doi:10.1016/S2542-5196(21)00285-0)
Supplement: Supplementary appendix [file mmc1.pdf]

THE LANCET
Planetary Health

Supplementary appendix

This appendix formed part of the original submission and has been peer reviewed. We post it as supplied by the authors.

Supplement to: Bai Y, Herforth A, Masters WA. Global variation in the cost of a nutrient-adequate diet by population group: an observational study. *Lancet Planet Health* 2022; **6:** e38–47.

Global variation in the cost of a nutrient adequate diet by population group: An observational study

Appendix of Supplementary Information

Yan Bai, Anna Herforth, and William A. Masters
Contact: william.masters@tufts.edu.
Project website: https://sites.tufts.edu/foodpricesfornutrition

Figure S1: **Flow chart of food item selection from the 2017 ICP dataset**
From 716 food and non-alcoholic beverage items in the ICP-2017 database, we excluded items that were non-caloric, specialized infant foods, or condiments whose quantity consumed would be negligible, and items whose size or composition was unknown, yielding a total of 545 food items to generate CoNA in 172 countries.

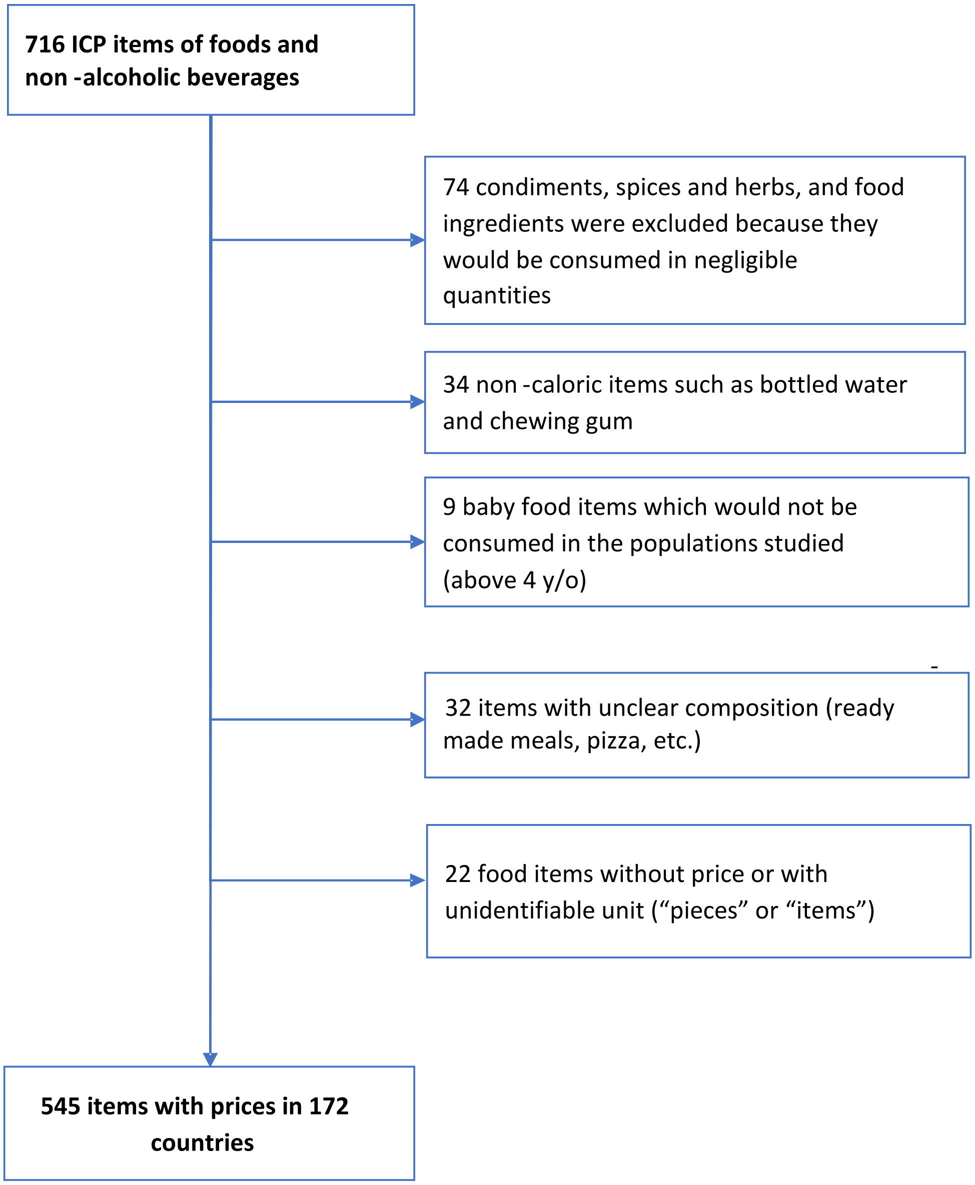

Figure S2: **Measured CoNA is higher in countries that report prices for very few foods**
CoNA is negatively correlated with the number of food items included in the ICP database for a country, with a Pearson's correlation coefficient of -0.3459 (P<0.001) and a Spearman's rho of -0.3026 (P<0.001).

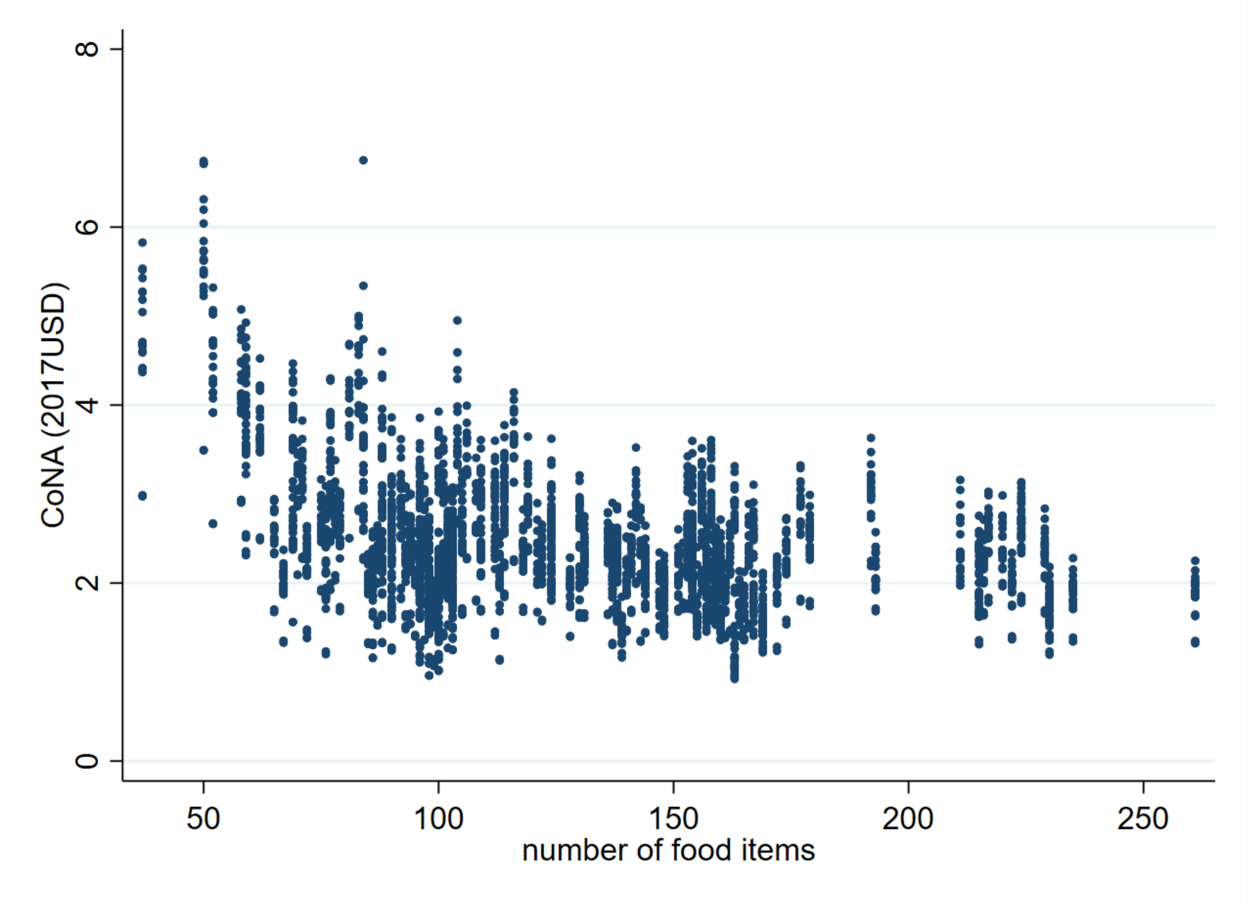

***Figure S3:* Composition of food groups in least-cost nutritious diets in selected countries**
(A) presents the energy portions; and (B) presents the weight portions. We selected 10 countries in the world to show the composition of food groups in least-cost nutritious diets at the country level.

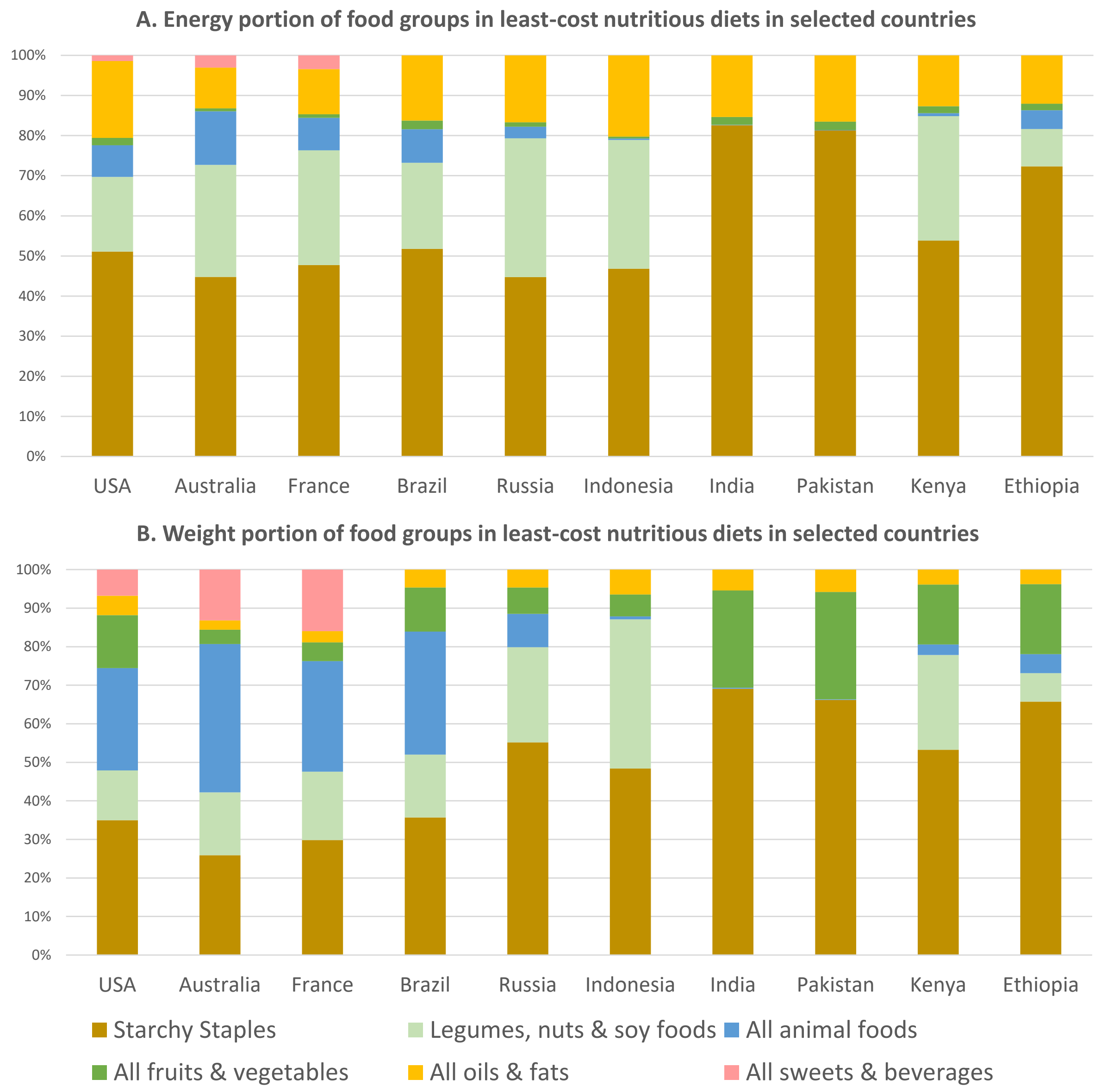

Table S1: Number of food items included in the price dataset for CoNA generation, by country and food group

N	Territories	Starchy staples	Pulses, nuts, and seeds	Animal-source foods	Fruits and vegetables	Oils & fats	Sweets and beverages	Total food items
1	Albania	21	4	31	16	6	15	93
2	Algeria	24	5	43	17	6	17	112
3	Angola	16	2	33	14	5	7	77
4	Antigua and Barbuda	13	3	23	18	5	8	70
5	Argentina	17	1	26	18	5	8	75
6	Armenia	44	7	75	44	11	48	229
7	Aruba	22	6	41	23	7	15	114
8	Australia	19	4	34	19	7	15	98
9	Austria	22	3	31	19	6	15	96
10	Azerbaijan	37	9	83	40	11	42	222
11	Bahamas, The	15	1	18	10	3	5	52
12	Bahrain	26	8	50	39	6	13	142
13	Bangladesh	48	8	73	25	14	25	193
14	Barbados	14	4	28	13	5	8	72
15	Belarus	40	8	75	40	9	44	216
16	Belgium	22	3	26	19	7	13	90
17	Belize	15	3	31	24	5	12	90
18	Benin	33	4	65	23	11	17	153
19	Bermuda	7	1	13	6	4	6	37
20	Bhutan	25	4	23	26	5	19	102
21	Bolivia	12	2	17	15	5	8	59
22	Bosnia and Herzegovina	21	4	29	19	5	14	92
23	Botswana	23	6	48	21	9	17	124
24	Brazil	18	3	34	20	6	11	92
25	British Virgin Islands	19	5	33	25	5	13	100
26	Brunei Darussalam	32	8	55	30	12	29	166
27	Bulgaria	23	4	37	19	7	15	105
28	Burkina Faso	32	5	63	24	12	18	154
29	Burundi	28	6	53	24	11	16	138
30	Cambodia	35	3	59	28	10	21	156
31	Cameroon	32	6	66	24	12	18	158
32	Canada	18	4	31	15	6	14	88
33	Cape Verde	29	6	52	24	8	17	136
34	Cayman Islands	23	6	42	25	7	15	118
35	Central African Republic	28	6	50	19	9	18	130
36	Chad	33	5	65	23	11	18	155
37	Chile	19	4	30	18	7	12	90
38	China	52	5	88	32	13	30	220

N	Territories	Starchy staples	Pulses, nuts, and seeds	Animal-source foods	Fruits and vegetables	Oils & fats	Sweets and beverages	Total food items
39	Colombia	16	3	24	16	7	13	79
40	Comoros	18	4	46	10	9	17	104
41	Congo, Dem. Rep.	31	6	65	24	12	18	156
42	Congo, Rep.	33	6	67	24	12	18	160
43	Costa Rica	12	2	23	11	6	11	65
44	Cote d'Ivoire	33	5	65	23	12	19	157
45	Croatia	23	3	34	19	7	15	101
46	Curacao	23	5	34	21	7	13	103
47	Cyprus	23	4	36	18	7	15	103
48	Czech Republic	21	3	36	18	6	13	97
49	Denmark	22	4	32	18	7	13	96
50	Djibouti	29	5	47	20	8	15	124
51	Dominica	17	3	30	20	6	12	88
52	Dominican Republic	21	6	39	25	7	14	112
53	Ecuador	15	3	22	20	5	11	76
54	Egypt, Arab Rep.	40	12	76	48	12	23	211
55	El Salvador	10	0	22	8	2	8	50
56	Equatorial Guinea	33	6	67	24	12	19	161
57	Estonia	21	3	33	18	7	14	96
58	Eswatini	30	6	52	24	9	17	138
59	Ethiopia	33	5	57	23	12	17	147
60	Fiji	20	4	34	17	5	17	97
61	Finland	21	2	28	17	5	13	86
62	France	23	4	36	17	6	14	100
63	Gabon	21	6	50	22	10	15	124
64	Gambia, The	33	6	66	24	12	19	160
65	Germany	22	3	31	18	7	13	94
66	Ghana	31	6	65	24	10	18	154
67	Greece	23	4	35	19	7	15	103
68	Grenada	16	4	15	22	7	13	77
69	Guinea	27	6	58	24	11	18	144
70	Guinea-Bissau	33	6	67	24	12	18	160
71	Guyana	22	6	40	16	7	15	106
72	Haiti	15	3	22	17	4	10	71
73	Honduras	11	3	21	18	3	6	62
74	Hong Kong SAR, China	29	5	74	34	10	22	174
75	Hungary	22	4	33	17	6	14	96
76	Iceland	19	3	29	18	6	13	88
77	India	54	10	92	34	14	31	235
78	Indonesia	34	3	70	32	10	28	177

N	Territories	Starchy staples	Pulses, nuts, and seeds	Animal-source foods	Fruits and vegetables	Oils & fats	Sweets and beverages	Total food items
79	Iran, Islamic Rep.	25	8	50	36	6	15	140
80	Iraq	31	10	59	44	8	15	167
81	Ireland	21	4	33	17	7	14	96
82	Israel	20	4	35	18	7	14	98
83	Italy	22	4	35	19	7	15	102
84	Jamaica	10	1	23	15	3	6	58
85	Japan	11	3	22	15	6	12	69
86	Jordan	31	10	61	44	8	15	169
87	Kazakhstan	54	10	84	52	12	49	261
88	Kenya	28	6	50	24	11	18	137
89	Korea, Rep.	14	3	29	17	6	14	83
90	Kuwait	32	10	57	42	8	15	164
91	Kyrgyz Republic	44	9	79	39	11	42	224
92	Lao PDR	32	3	64	28	4	25	156
93	Latvia	22	4	34	19	7	15	101
94	Lesotho	25	4	48	20	8	16	121
95	Liberia	30	6	50	21	7	17	131
96	Lithuania	23	4	33	18	7	13	98
97	Luxembourg	23	3	35	19	7	14	101
98	Madagascar	26	5	45	21	7	15	119
99	Malawi	33	5	59	24	8	19	148
100	Malaysia	27	5	61	31	11	27	162
101	Maldives	12	1	14	20	9	13	69
102	Mali	33	6	65	24	12	19	159
103	Malta	23	4	35	19	7	15	103
104	Mauritania	29	6	47	21	10	17	130
105	Mauritius	28	6	58	24	9	18	143
106	Mexico	20	4	33	17	6	14	94
107	Moldova	46	9	85	37	11	42	230
108	Mongolia	37	4	38	28	11	26	144
109	Montenegro	20	3	34	19	5	14	95
110	Morocco	47	13	87	47	11	25	230
111	Mozambique	33	6	65	24	11	19	158
112	Myanmar	53	8	81	33	13	29	217
113	Namibia	30	5	48	21	8	16	128
114	Nepal	35	7	35	24	7	23	131
115	Netherlands	23	4	31	19	7	14	98
116	New Zealand	16	3	24	13	5	11	72
117	Nicaragua	14	2	24	24	5	9	78
118	Niger	30	5	65	23	11	18	152
119	Nigeria	32	6	64	24	12	19	157

N	Territories	Starchy staples	Pulses, nuts, and seeds	Animal-source foods	Fruits and vegetables	Oils & fats	Sweets and beverages	Total food items
120	North Macedonia	21	3	30	16	5	13	88
121	Norway	20	2	29	17	6	13	87
122	Oman	31	10	60	43	8	15	167
123	Pakistan	52	10	79	31	12	31	215
124	Panama	18	2	27	19	7	11	84
125	Paraguay	18	2	27	20	6	8	81
126	Peru	23	6	36	23	7	14	109
127	Philippines	33	6	81	31	12	29	192
128	Poland	21	4	34	19	7	15	100
129	Portugal	22	4	33	19	7	14	99
130	Qatar	32	10	59	41	6	15	163
131	Romania	21	4	36	18	6	15	100
132	Russian Federation	18	4	30	16	5	13	86
133	Rwanda	30	5	55	22	10	17	139
134	Sao Tome and Principe	28	6	42	24	7	15	122
135	Saudi Arabia	32	10	60	44	8	15	169
136	Senegal	33	6	67	23	12	18	159
137	Serbia	22	4	35	19	7	15	102
138	Seychelles	33	4	52	22	9	17	137
139	Sierra Leone	32	6	60	24	12	19	153
140	Singapore	33	3	67	31	12	26	172
141	Sint Maarten (Dutch part)	12	2	21	11	5	8	59
142	Slovak Republic	20	4	37	19	6	14	100
143	Slovenia	23	4	35	19	7	15	103
144	South Africa	15	2	33	17	5	12	84
145	Spain	23	4	32	19	7	15	100
146	Sri Lanka	28	7	45	29	11	18	138
147	St. Kitts and Nevis	21	6	36	24	7	15	109
148	St. Lucia	20	6	36	25	6	15	108
149	St. Vincent and the Grenadines	19	4	32	24	7	10	96
150	Sudan	30	10	56	36	9	13	154
151	Suriname	24	6	39	25	7	15	116
152	Sweden	18	4	29	15	6	13	85
153	Switzerland	23	3	31	19	6	14	96
154	Taiwan	25	2	64	30	11	26	158
155	Tajikistan	42	9	71	31	14	48	215
156	Tanzania	32	6	66	24	12	19	159
157	Thailand	30	3	62	30	10	28	163
158	Togo	24	3	44	8	8	11	98
159	Trinidad and Tobago	24	6	38	25	7	14	114

N	Territories	Starchy staples	Pulses, nuts, and seeds	Animal-source foods	Fruits and vegetables	Oils & fats	Sweets and beverages	Total food items
160	Tunisia	20	6	46	18	7	16	113
161	Turkey	21	4	30	18	7	13	93
162	Turks and Caicos Islands	21	5	37	24	6	12	105
163	Uganda	33	6	59	23	11	19	151
164	United Arab Emirates	28	10	61	44	7	15	165
165	United Kingdom	23	3	33	18	7	15	99
166	United States	14	3	30	15	5	9	76
167	Uruguay	15	5	21	12	6	8	67
168	Uzbekistan	39	7	68	45	12	53	224
169	Vietnam	37	5	72	30	9	26	179
170	West Bank and Gaza	27	8	55	39	6	13	148
171	Zambia	29	5	56	24	9	18	141
172	Zimbabwe	32	6	61	24	11	19	153
	**Average**	**26**	**5**	**46**	**23**	**8**	**17**	**125**

Table S2: Dietary Reference Intakes applied in the calculation of CoNA

DRIs	Description	Nutrients
EAR	Estimated average requirement to meet the needs of at least 50% of a healthy population in each demographic group[1]	Protein, carbohydrates, calcium, magnesium, phosphorus, copper, selenium, vitamin C, thiamin, riboflavin, niacin, vitamin B6, folate, vitamin B12, vitamin A, vitamin E
RDA	Recommended dietary allowance to meet the needs of at least 97.5% of a healthy population in each demographic group[1]	Protein, carbohydrates, calcium, iron, magnesium, phosphorus, zinc, copper, selenium, vitamin C, thiamin, riboflavin, niacin, vitamin B6, folate, vitamin B12, vitamin A, vitamin E
H-AR[4]	Harmonized average requirements to meet the needs of at least 50% of a healthy population in each demographic group, in settings with low bioavailability[2]	Iron and zinc
UL[5]	Upper levels to avoid toxicity and pose no risk of adverse health effects in a healthy population[1]	Calcium, iron, phosphorus, zinc, copper, selenium, vitamin C, vitamin B6, vitamin A (retinol)
CDRR	Chronic disease risk reduction level of intake to limit chronic disease risk in a healthy population.[3]	Sodium
AMDR	Acceptable macronutrient distribution ranges within which there is a reduced risk of chronic disease.[1]	Protein, carbohydrates and lipids

Notes:
1. Institute of Medicine (IOM). 2006.
2. Allen et al., 2019
3. National Academies of Sciences, Engineering, and Medicine. 2019.
4. We used the requirement for iron with a low absorption assumption, and for zinc of a “semi-unrefined diet”.
5. UL of magnesium, niacin, folate and vitamin E refer to pharmacological agent or supplements, and therefore are excluded from our linear programing constraints

Table S3: CoNA per day in 2017USD over 20 demographic groups and 4 country income levels using EARs/H-ARs

No.	Categories	Countries, n	Mean	Standard Deviation	25th Percentile	Median	75th Percentile
	**Demographic groups**						
	**Male**						
1	4-8y	172	1.72	0.43	1.41	1.67	1.94
2	9-13y	172	2.67	0.81	2.11	2.51	2.94
3	14-18y	172	2.87	0.77	2.31	2.72	3.15
4	19-30y	172	2.42	0.65	1.98	2.31	2.73
5	31-50y	172	2.39	0.65	1.96	2.26	2.69
6	51-70y	172	2.37	0.65	1.93	2.24	2.68
7	70y+	172	2.55	0.68	2.07	2.40	2.88
	**Female**						
8	4-8y	172	1.70	0.43	1.39	1.64	1.92
9	9-13y	172	2.50	0.72	1.98	2.34	2.79
10	14-18y	172	2.60	0.73	2.08	2.44	2.90
11	19-30y	172	2.26	0.67	1.83	2.14	2.54
12	31-50y	172	2.25	0.67	1.82	2.12	2.53
13	51-70y	172	2.39	0.65	1.92	2.26	2.67
14	70y+	172	2.39	0.66	1.92	2.24	2.66
	**Female, pregnancy**						
15	14-18y	172	2.66	0.71	2.18	2.49	2.96
16	19-30y	172	2.47	0.67	2.04	2.34	2.81
17	31-50y	172	2.45	0.68	2.01	2.33	2.80
	**Female, lactation**						
18	14-18y	172	2.82	0.80	2.29	2.64	3.15
19	19-30y	172	2.65	0.78	2.14	2.49	3.01
20	31-50y	172	2.64	0.78	2.13	2.48	3.00
	**Country income levels**						
1	High income	1,260	2.35	0.80	1.85	2.17	2.64
2	Upper-middle income	880	2.63	0.69	2.18	2.56	2.99
3	Lower-middle income	760	2.51	0.76	2.03	2.39	2.80
4	Low income	540	2.22	0.55	1.82	2.15	2.45
	**Total**	3,440	2.44	0.74	1.95	2.32	2.76

Note: Data shown are reported in USD at PPP exchange rates for 2017, using EARs and H-ARs (for iron and zinc) to reflect an adequate level of intake for at least 50% of a healthy population. Each country in the analysis has 20 demographic groups, or 20 observations, so there are 63 high-income, 44 upper-middle income, 38 lower-middle, and 29 low-income countries.

Table S4: CoNA per day in 2017USD over 20 demographic groups and 4 country income levels using RDAs

No.	Categories	Countries, n	Mean	Standard Deviation	25th Percentile	Median	75th Percentile
	**Demographic groups**						
	**Male**						
1	4-8y	172	2.05	0.51	1.71	1.98	2.32
2	9-13y	172	3.05	0.91	2.44	2.86	3.43
3	14-18y	172	3.26	0.87	2.64	3.06	3.61
4	19-30y	172	2.79	0.74	2.29	2.63	3.11
5	31-50y	172	2.77	0.75	2.27	2.60	3.08
6	51-70y	172	2.76	0.75	2.26	2.60	3.10
7	70y+	172	2.98	0.79	2.47	2.80	3.29
	**Female**						
8	4-8y	172	2.03	0.51	1.69	1.97	2.30
9	9-13y	172	2.90	0.83	2.35	2.71	3.26
10	14-18y	172	3.02	0.86	2.45	2.81	3.32
11	19-30y	172	2.64	0.78	2.14	2.45	2.90
12	31-50y	172	2.64	0.79	2.13	2.45	2.91
13	51-70y	172	2.83	0.78	2.38	2.60	3.13
14	70y+	172	2.84	0.80	2.38	2.60	3.18
	**Female, pregnancy**						
15	14-18y	172	3.31	0.92	2.72	3.14	3.59
16	19-30y	172	2.96	0.84	2.42	2.80	3.29
17	31-50y	172	2.96	0.85	2.41	2.77	3.26
	**Female, lactation**						
18	14-18y	172	3.45	0.96	2.83	3.22	3.85
19	19-30y	172	3.10	0.89	2.54	2.93	3.45
20	31-50y	172	3.10	0.90	2.54	2.94	3.44
	**Country income levels**						
1	High income	1,260	2.75	0.94	2.16	2.54	3.06
2	Upper-middle income	880	3.08	0.82	2.55	2.96	3.50
3	Lower-middle income	760	2.96	0.91	2.39	2.84	3.29
4	Low income	540	2.69	0.65	2.24	2.59	2.98
	**Total**	3,440	2.87	0.88	2.31	2.72	3.24

Note: Data shown are reported in USD at PPP exchange rates for 2017, using RDAs to reflect an adequate level of intake for at least 97.5% of a healthy population. Each country in the analysis has 20 demographic groups, or 20 observations, so there are 63 high-income, 44 upper-middle income, 38 lower-middle, and 29 low-income countries.

Table S5: CoNA per 1,000kcal in 2017USD over 20 demographic groups and 4 country income levels using EARs and H-ARs

No.	Categories	Countries, n	Mean	Standard Deviation	25th Percentile	Median	75th Percentile
	**Demographic groups**						
	**Male**						
1	4-8y	172	0.95	0.24	0.77	0.92	1.06
2	9-13y	172	1.11	0.34	0.88	1.05	1.23
3	14-18y	172	0.90	0.24	0.73	0.86	0.99
4	19-30y	172	0.82	0.22	0.67	0.78	0.93
5	31-50y	172	0.85	0.23	0.70	0.81	0.96
6	51-70y	172	0.91	0.25	0.74	0.86	1.03
7	70y+	172	1.03	0.27	0.84	0.97	1.17
	**Female**						
8	4-8y	172	1.00	0.25	0.82	0.97	1.13
9	9-13y	172	1.17	0.34	0.93	1.09	1.30
10	14-18y	172	1.09	0.31	0.87	1.03	1.22
11	19-30y	172	0.95	0.28	0.77	0.90	1.07
12	31-50y	172	1.00	0.30	0.81	0.94	1.12
13	51-70y	172	1.13	0.31	0.90	1.06	1.26
14	70y+	172	1.18	0.33	0.95	1.11	1.32
	**Female, pregnancy**						
15	14-18y	172	0.95	0.25	0.77	0.88	1.05
16	19-30y	172	0.86	0.23	0.71	0.82	0.98
17	31-50y	172	0.89	0.25	0.73	0.84	1.02
	**Female, lactation**						
18	14-18y	172	1.06	0.30	0.86	0.99	1.18
19	19-30y	172	0.98	0.29	0.79	0.92	1.12
20	31-50y	172	1.02	0.30	0.82	0.96	1.16
	**Country income levels**						
1	High income	1,260	0.95	0.32	0.75	0.87	1.07
2	Upper-middle income	880	1.07	0.27	0.88	1.03	1.21
3	Lower-middle income	760	1.03	0.31	0.83	0.98	1.14
4	Low income	540	0.91	0.24	0.74	0.88	1.02
	**Total**	3,440	0.99	0.30	0.80	0.94	1.12

Note: Data shown are reported in USD at PPP exchange rates for 2017, using EARs and H-ARs (for iron and zinc) to reflect an adequate level of intake for at least 50% of a healthy population. Each country in the analysis has 20 demographic groups, or 20 observations, so there are 63 high-income, 44 upper-middle income, 38 lower-middle, and 29 low-income countries.

Table S6: CoNA per 1,000kcal in 2017USD over 20 demographic groups and 4 country income levels using RDAs

No.	Categories	Countries, n	Mean	Standard Deviation	25th Percentile	Median	75th Percentile
	**Demographic groups**						
	**Male**						
1	4-8y	172	1.13	0.28	0.94	1.09	1.28
2	9-13y	172	1.27	0.38	1.02	1.19	1.43
3	14-18y	172	1.03	0.27	0.83	0.96	1.14
4	19-30y	172	0.95	0.25	0.78	0.89	1.05
5	31-50y	172	0.99	0.27	0.81	0.93	1.10
6	51-70y	172	1.06	0.29	0.87	1.00	1.19
7	70y+	172	1.21	0.32	1.00	1.14	1.34
	**Female**						
8	4-8y	172	1.20	0.30	0.99	1.16	1.36
9	9-13y	172	1.36	0.39	1.10	1.27	1.53
10	14-18y	172	1.27	0.36	1.03	1.18	1.40
11	19-30y	172	1.12	0.33	0.90	1.03	1.23
12	31-50y	172	1.17	0.35	0.94	1.08	1.29
13	51-70y	172	1.34	0.37	1.12	1.23	1.48
14	70y+	172	1.41	0.39	1.18	1.29	1.57
	**Female, pregnancy**						
15	14-18y	172	1.18	0.33	0.97	1.12	1.28
16	19-30y	172	1.03	0.29	0.85	0.98	1.15
17	31-50y	172	1.07	0.31	0.88	1.01	1.18
	**Female, lactation**						
18	14-18y	172	1.29	0.36	1.06	1.21	1.44
19	19-30y	172	1.15	0.33	0.94	1.09	1.28
20	31-50y	172	1.20	0.35	0.98	1.14	1.33
	**Country income levels**						
1	High income	1,260	1.12	0.37	0.88	1.02	1.24
2	Upper-middle income	880	1.26	0.32	1.03	1.20	1.41
3	Lower-middle income	760	1.21	0.37	0.98	1.15	1.34
4	Low income	540	1.10	0.29	0.90	1.07	1.23
	**Total**	3,440	1.17	0.35	0.94	1.10	1.31

Note: Data shown are reported in USD at PPP exchange rates for 2017, using RDAs to reflect an adequate level of intake for at least 97.5% of a healthy population. Each country in the analysis has 20 demographic groups, or 20 observations, so there are 63 high-income, 44 upper-middle income, 38 lower-middle, and 29 low-income countries.

Table S7: Regression results on cost of diet and cost of diet per 1,000kcal across demographic groups

		Cost of diet				Cost of diet per 1,000kcal			
**N**	**Categorical variables**	**Beta coef**	**P-value**	**95% CI**		**Beta coef**	**P-value**	**95% CI**	
	**Sex-physiological group**								
1	Male	(base)				(base)			
2	Female	(0.16)	0.00	(0.18)	(0.13)	0.13	0.00	0.12	0.15
3	Pregnancy	0.05	0.00	0.03	0.08	0.04	0.00	0.03	0.06
4	Lactation	0.24	0.00	0.20	0.27	0.16	0.00	0.15	0.18
	**Age group**								
5	4-8y	(0.69)	0.00	(0.74)	(0.64)	0.13	0.00	0.11	0.15
6	9-13y	0.25	0.00	0.19	0.31	0.29	0.00	0.27	0.32
7	14-18y	0.45	0.00	0.42	0.48	0.08	0.00	0.07	0.10
8	19-30y	(base)				(base)			
9	31-50y	(0.03)	0.09	(0.06)	0.00	0.04	0.00	0.02	0.05
10	51-70y	(0.05)	0.00	(0.08)	(0.02)	0.09	0.00	0.08	0.10
11	70y+	0.13	0.00	0.11	0.16	0.21	0.00	0.20	0.23
	**Sex-physiological group # age group**								
12	Female#4-8y	0.13	0.00	0.06	0.20	(0.08)	0.00	(0.11)	(0.06)
13	Female#9-13y	(0.01)	0.81	(0.08)	0.06	(0.08)	0.00	(0.11)	(0.05)
14	Female#14-18y	(0.11)	0.00	(0.15)	(0.07)	0.05	0.00	0.04	0.07
15	Female#31-50y	0.02	0.35	(0.02)	0.05	0.01	0.42	(0.01)	0.02
16	Female#51-70y	0.18	0.00	0.14	0.21	0.08	0.00	0.07	0.10
17	Female#70y+	(0.01)	0.71	(0.04)	0.03	0.01	0.12	(0.00)	0.03
18	Pregnancy#14-18y	(0.26)	0.00	(0.30)	(0.22)	(0.00)	0.95	(0.02)	0.02
19	Pregnancy#31-50y	0.01	0.67	(0.03)	0.05	(0.01)	0.48	(0.03)	0.01
20	Lactation#14-18y	(0.28)	0.00	(0.33)	(0.23)	(0.01)	0.44	(0.03)	0.01
21	Lactation#31-50y	0.02	0.57	(0.04)	0.07	0.00	0.82	(0.02)	0.02

Note: Data shown are GLM regression results, adjusted for countries' fixed effects, using CoNA based on EARs and H-AR levels that meet requirements for at least 50% of a healthy population. The parentheses indicate a negative sign.

Table S8: Composition of least-cost diets by demographic group and income level (g/day)

No.	Categories	Countries, n	Starchy staples	Pulses, nuts and seeds	Animal-source foods	Fruits and vegetables	All fats and oils	Sweets and beverages
	**Demographic groups**							
	**Male**							
1	4-8y	172	308	99	189	73	30	15
2	9-13y	172	355	215	220	132	38	15
3	14-18y	172	539	203	212	150	57	28
4	19-30y	172	532	163	146	145	52	30
5	31-50y	172	503	161	146	149	47	29
6	51-70y	172	473	161	149	151	42	23
7	70y+	172	442	162	208	170	34	22
	**Female**							
8	4-8y	172	287	99	190	75	27	12
9	9-13y	172	325	192	247	125	25	13
10	14-18y	172	406	177	226	148	31	19
11	19-30y	172	426	168	115	126	38	21
12	31-50y	172	403	169	114	129	35	20
13	51-70y	172	386	149	207	154	28	16
14	70y+	172	364	148	207	159	26	16
	**Female, pregnancy**							
15	14-18y	172	522	184	187	142	42	20
16	19-30y	172	537	180	132	131	48	21
17	31-50y	172	518	179	132	133	45	20
	**Female, lactation**							
18	14-18y	172	483	181	201	214	41	26
19	19-30y	172	490	179	147	203	46	29
20	31-50y	172	469	180	148	205	43	26
	**Averages by country income level**							
1	High income	63	444	142	260	101	39	39
2	Upper-middle income	44	472	138	149	159	47	16
3	Lower-middle income	38	432	163	99	179	39	6
4	Low income	27	378	279	132	182	24	8
	**Total**	172	438	167	176	146	39	21

Note: Data shown are based on EARs and H-ARs that meet requirements for at least 50% of a healthy population. Averages by country income level are the mean over the 20 demographic groups in the number of countries shown, not weighted by population size or age-sex composition.

Table S9: Composition of least-cost diets by demographic group and income level (kcal/day)

No.	Categories	Total energy	Countries, n	Starchy staples	Pulses, nuts and seeds	Animal-source foods	Fruits and vegetables	All fats and oils	Sweets and beverages
	**Demographic groups**								
	**Male**								
1	4-8y	1,821	172	951	328	202	33	261	45
2	9-13y	2,395	172	1,036	711	236	55	325	32
3	14-18y	3,175	172	1,632	701	211	75	495	61
4	19-30y	2,947	172	1,654	585	139	71	445	53
5	31-50y	2,795	172	1,552	578	140	71	407	48
6	51-70y	2,604	172	1,415	579	144	71	360	36
7	70y+	2,466	172	1,281	570	213	74	292	37
	**Female**								
8	4-8y	1,698	172	869	327	204	33	231	34
9	9-13y	2,139	172	945	646	259	52	214	24
10	14-18y	2,377	172	1,172	607	234	63	270	32
11	19-30y	2,367	172	1,241	587	121	58	325	35
12	31-50y	2,256	172	1,157	589	121	59	300	31
13	51-70y	2,118	172	1,066	507	211	65	243	27
14	70y+	2,018	172	991	499	212	65	226	24
	**Female, pregnancy**								
15	14-18y	2,842	172	1,519	639	188	66	363	38
16	19-30y	2,865	172	1,588	639	128	64	411	35
17	31-50y	2,754	172	1,511	633	129	64	385	32
	**Female, lactation**								
18	14-18y	2,664	172	1,344	636	199	90	359	36
19	19-30y	2,697	172	1,385	645	141	88	397	42
20	31-50y	2,586	172	1,308	646	143	89	368	32
	**Averages by country income level**								
1	High income		63	1,351	474	224	52	344	33
2	Upper-middle income		44	1,324	466	145	79	402	62
3	Lower-middle income		38	1,333	577	154	66	326	22
4	Low income		27	972	1,034	162	73	212	25
	**Total**		172	1,281	583	179	65	334	37

Note: Data shown under total energy is estimated average requirements (EER) with active levels of physical activity for each population group. Diets are based on EARs and H-ARs that meet requirements for at least 50% of a healthy population. Averages by country income level are the mean over the 20 demographic groups in the number of countries shown, not weighted by population size or age-sex composition.

Table S10: Differences in composition of least-cost diets by national income levels

	Intake (gram)				Energy (kcal)			
Categorical variables	Beta coef	P-value	95% CI		Beta coef	P-value	95% CI	
**Starchy staples**								
Upper-middle income	27.8	0.00	8.9	46.6	(27.4)	0.24	(72.7)	17.9
Lower-middle income	(12.3)	0.17	(29.9)	5.4	(18.2)	0.48	(68.3)	31.8
Low income	(66.4)	0.00	(92.7)	(40.1)	(378.9)	0.00	(442.8)	(315.0)
**Pulses, nuts and seeds**								
Upper-middle income	2.3	0.62	(6.8)	11.4	(3.1)	0.85	(34.9)	28.6
Lower-middle income	14.6	0.02	2.1	27.2	98.0	0.00	50.2	145.7
Low income	146.2	0.00	126.2	166.1	567.3	0.00	504.3	630.4
**Animal-source foods**								
Upper-middle income	(117.1)	0.00	(130.8)	(103.4)	(84.3)	0.00	(94.7)	(73.8)
Lower-middle income	(155.0)	0.00	(168.3)	(141.7)	(65.4)	0.00	(80.5)	(50.2)
Low income	(137.6)	0.00	(155.2)	(120.0)	(69.9)	0.00	(85.8)	(53.9)
**Fruits and vegetables**								
Upper-middle income	57.7	0.00	44.2	71.1	27.4	0.00	17.8	37.1
Lower-middle income	78.0	0.00	61.2	94.7	13.9	0.00	5.4	22.4
Low income	81.2	0.00	54.1	108.4	20.6	0.00	7.7	33.6
**Oils and fats**								
Upper-middle income	8.3	0.00	6.5	10.0	58.2	0.00	46.2	70.2
Lower-middle income	0.3	0.79	(1.9)	2.5	(17.6)	0.03	(32.9)	(2.2)
Low income	(15.1)	0.00	(17.0)	(13.1)	(131.9)	0.00	(149.1)	(114.8)
**Sweets and beverages**								
Upper-middle income	(22.9)	0.00	(27.9)	(17.9)	29.1	0.00	16.6	41.6
Lower-middle income	(33.4)	0.00	(37.9)	(28.9)	(10.7)	0.02	(19.7)	(1.7)
Low income	(31.1)	0.00	(36.4)	(25.8)	(7.3)	0.21	(18.8)	4.2

Note: Data shown are GLM regression results, adjusted for each of 20 demographic groups, using CoNA based on EARs and H-AR levels that meet requirements for at least 50% of a healthy population. In the regression models, the reference group for comparisons is the average intake in the high-income countries. The parentheses indicate a negative sign.

Table S11: Sensitivity of diet costs to binding nutrient constraints

No.	Nutrients	High income	Upper-middle income	Lower-middle income	Low income	All countries
	**Energy and Macronutrients**					
1	Energy	0.17	0.24	0.31	0.20	0.22
2	Protein, AMDR, lower	0.00	0.01	0.01	0.00	0.00
3	Lipid, AMDR, lower	0.02	0.01	0.01	0.01	0.02
4	Lipid, AMDR, upper	(0.01)	(0.01)	(0.01)	(0.03)	(0.01)
5	Carbs, AMDR, lower	0.00	0.00	0.00	0.01	0.00
6	Carbs, AMDR, upper	(0.10)	(0.12)	(0.13)	(0.04)	(0.10)
	**Minerals**					
7	Calcium, EAR	0.32	0.36	0.49	0.65	0.42
8	Iron, H-AR	0.23	0.18	0.12	0.12	0.17
9	Magnesium, EAR	0.01	0.01	0.01	0.00	0.01
10	Zinc, H-AR	0.05	0.13	0.08	0.03	0.07
11	Copper, UL	0.00	0.00	0.00	(0.01)	0.00
12	Selenium, UL	0.00	0.00	0.00	(0.01)	0.00
13	Sodium, UL	(0.03)	(0.07)	(0.08)	(0.08)	(0.06)
	**Vitamins**					
14	Vitamin C, EAR	0.10	0.08	0.07	0.06	0.08
15	Riboflavin, EAR	0.02	0.02	0.01	0.00	0.01
16	Niacin, EAR	0.06	0.03	0.01	0.01	0.04
17	Vitamin B6, EAR	0.04	0.02	0.02	0.00	0.02
18	Folate, EAR	0.01	0.00	0.01	0.00	0.01
19	Vitamin B12, EAR	0.04	0.04	0.01	0.01	0.03
20	Vitamin A, EAR	0.03	0.02	0.02	0.02	0.02
21	Vitamin E, EAR	0.04	0.07	0.06	0.05	0.05

Note: Data shown are simple averages, not weighted by population size, over all demographic groups and countries in each national income category. Diet costs are based on EARs and H-ARs that meet requirements for at least 50% of a healthy population. Sensitivity is measured as the shadow price elasticity of each constraint, defined as the percent change in cost per day for each one percent change in the lower or upper bound, shown here in absolute value of the ratio (for example, a 1 percent increase in energy requirements would raise diet costs by 0.15 percent.) Nutrient constraints whose average magnitude of shadow price elasticity is below 0.001 for all income levels are not shown. The parentheses indicate a negative sign.

Table S12: Differences in sensitivity of diet costs to nutrient constraints, by income level

	Shadow price elasticities							
**Categorical variables**	**Beta coef**	**P-value**	**95% CI**		**Beta coef**	**P-value**	**95% CI**	
	**Estimated Energy Requirement**				**Zinc, EAR**			
Upper-middle income	0.06	0.00	0.04	0.09	0.08	0.00	0.06	0.09
Lower-middle income	0.14	0.00	0.11	0.17	0.03	0.00	0.01	0.04
Low income	0.02	0.11	(0.01)	0.05	(0.02)	0.00	(0.03)	(0.01)
	**Carbohydrate, AMDR (upper bound)**				**Sodium, CDRR (upper limit)**			
Upper-middle income	0.02	0.00	0.01	0.03	0.04	0.00	0.03	0.06
Lower-middle income	0.03	0.00	0.01	0.04	0.04	0.00	0.03	0.06
Low income	(0.06)	0.00	(0.07)	(0.05)	0.05	0.00	0.03	0.06
	**Calcium, EAR**				**Vitamin C, EAR**			
Upper-middle income	0.03	0.00	0.02	0.05	(0.02)	0.00	(0.02)	(0.02)
Lower-middle income	0.16	0.00	0.15	0.18	(0.03)	0.00	(0.03)	(0.03)
Low income	0.32	0.00	0.30	0.35	(0.04)	0.00	(0.04)	(0.03)
	**Iron, H-AR**				**Vitamin E, EAR**			
Upper-middle income	(0.05)	0.00	(0.07)	(0.03)	0.03	0.00	0.02	0.04
Lower-middle income	(0.10)	0.00	(0.12)	(0.08)	0.02	0.00	0.01	0.03
Low income	(0.11)	0.00	(0.13)	(0.08)	0.01	0.09	(0.00)	0.01

Note: Data shown are GLM regression results, adjusted for each of 20 demographic groups, using CoNA based on EARs and H-AR levels that meet requirements for at least 50% of a healthy population. The dependent variable is shadow price elasticities as defined in Table S11, showing results for the eight nutrients to which diet costs are most sensitive. For the comparability purpose, we used the magnitude (absolute value) of shadow price elasticities for the upper bound limits of carbohydrate (AMDR) and sodium (CDRR). The parentheses indicate a negative sign.
